# Plasmablast, memory B cell and T follicular helper cell responses after human papillomavirus vaccination: effect of dose number and age

**DOI:** 10.1038/s41541-026-01408-w

**Published:** 2026-02-21

**Authors:** Eunice W. Kiamba, Dolapo O. Ajiboye, Adedapo Olufemi Bashorun, Mamie Ndeban Jallow, Lamin Drammeh, Samba Bah, Tijan Jobarteh, Francis Kanu, Ousubie Jawla, Jobarteh Lamin, Anne Segonds-Pichon, Martin J. Holland, Martin R. Goodier, Sophie Roetynck, Ed Clarke

**Affiliations:** 1 Vaccines and Immunity Theme, MRC Unit The Gambia at London School of Hygiene and Tropical Medicine, Banjul, Gambia; 2 Department of Clinical Research, London School of Hygiene and Tropical Medicine, London, UK; 3 Edward Francis Small Teaching Hospital, Banjul, Gambia; 4 Department of Infection Biology, London School of Hygiene and Tropical Medicine, London, UK; 5 Disease Control and Elimination Theme, MRC Unit The Gambia at London School of Hygiene and Tropical Medicine, Banjul, Gambia

**Keywords:** Cancer, Immunology, Oncology

## Abstract

Multiple doses of HPV vaccines induce durable, antibody-mediated protection against HPV infections and HPV-associated diseases. Although actual protection against disease by a single HPV vaccination dose has not been confirmed in randomised trials, this regimen induces protection against incident and persistent HPV infection, similar to multi-dose schedules. However, the cellular mechanisms driving durable antibody responses to subunit vaccines remain poorly understood. B cells and T follicular helper (Tfh) cells play central roles in long-term antibody-mediated immunity. We characterised plasmablast, memory B cell (Bmem), and Tfh cell responses to assess the effects of dose number and age following HPV vaccination in Gambian females aged 4–26 years. A significant induction of HPV16/18-specific IgM plasmablasts occurred after the first dose, while robust HPV16/18-specific IgG plasmablast, Bmem, and Tfh responses were observed after two or three doses. Activation within the total Tfh pool increased with decreasing age, whereas HPV16/18-specific Tfh activation was higher in older vaccinees. These findings demonstrate the potential of multi-dose HPV vaccination schedules to sustain antibody protection through coordinated B cell and Tfh responses and highlight the need for continued monitoring of single-dose regimen. Exploring HPV vaccination in children under nine years may improve delivery and uptake.

## Introduction

Cervical cancer, the fourth most common cause of cancer deaths in women globally, is almost universally associated with persistent infection with certain ‘high risk’ types of HPV^1,2^. Eight licensed HPV vaccines are highly efficacious against the HPV types contained in each vaccine with some cross-protection against some non-vaccine HPV types^3,4^. It has been broadly demonstrated that HPV vaccines generate durable antibody-mediated protection against the HPV types they contain, with antibody titre sustained without waning for nearly 20 years for the quadrivalent (Gardasil), 12 years for the nine-valent (Gardasil 9), and up to 16 years for the bivalent (Cervarix) vaccine as of the time of writing^5–10^. However, although antibodies protect against infection, there is no established correlate of protection for HPV vaccines due to their high efficacy against infection. Additionally, due to the long natural history of HPV-associated cervical cancer (10–30 years), studies to determine a correlate of protection against the disease would require complex long-term studies, which would be ethically constrained as not feasible to conduct a decades-long randomised trial with a placebo group until cancer development.

Although antibody titre induced by a single HPV vaccination dose is usually significantly below those induced by multiple-dose regimens, they are similarly sustained at several folds above naturally induced titre and are highly protective against persistent HPV infection^5,11–13^. A recent post hoc analysis of five phase 3 trials of the Gardasil 9 vaccine reported contrasting findings, that a single dose of the vaccine induced antibody levels that are similar to or lower than naturally induced levels, which may suggest variability across studies and populations^14^. Such variations may be expected from different studies due to various potential confounders especially if no standardised methodology was used across the analysed trials. Based on the available evidence on vaccination immunogenicity and efficacy, WHO recommended a single dose HPV vaccination in girls and young women aged between 9 and 20 years as an alternative to the labelled dosing schedule^15^. Importantly, WHO also recommended research priorities for generating further evidence on long-term immunogenicity, efficacy, effectiveness and duration of protection of single-dose HPV schedules in girls, women, boys and men within the currently targeted age groups, as well as exploring vaccination in children under nine years of age. Subsequently, several randomised trials were initiated to evaluate the efficacy and immunogenicity of the single-dose HPV vaccination. The first single-dose randomised controlled efficacy trial was performed in Kenya, recruiting females aged 15–20 years^16,17^. This study reported the efficacy of a single dose of Cervarix and Gardasil 9 against new HPV 16/18 infections, with efficacy of about 98% three years after vaccination. Another randomised-controlled trial reported stable antibody responses for up to five years following a single dose of Cervarix or Gardasil 9 in Tanzanian girls aged 9–14 years^12^. Similar to reports from observational studies in India and Costa Rica, this trial showed that the single-dose antibody responses are maintained at levels below those generated by 2 and 3 doses.

The ESCUDDO trial has recently demonstrated non-inferiority of a single dose compared to two doses for both Cervarix and Gardasil 9 vaccines, in the prevention of persistent infection with HPV 16/18 in girls aged between 12 and 16 years^18,19^. The ESCUDDO trial also reported vaccine effectiveness of at least 97% by comparing HPV 16/18 infection among the trial participants with that among girls and women enroled in a non-randomised survey. Other ongoing randomised trials are also expected to provide further evidence for efficacy, immunogenicity and durability of the single dose schedule.

It is important that vaccine immunogenicity is evaluated comprehensively, including antibody responses and the early corresponding cellular immune responses to guide vaccination policies^20^. Such comprehensive characterisation of immunity to highly effective vaccines such as HPV vaccines may provide useful insights for the development of similarly effective vaccines to other antigens. Evaluation of long-term cellular immunity includes assessing cell populations such as Bmem and Tfh cells, which play a critical role in generating anamnestic responses to boost and maintain antibody titre for continued protection^21^. Long-lived plasma cells (LLPCs) play a key role in the maintenance of long-term antibody responses against different antigens^22^. However, the homing of LLPCs in specialised niches in the bone marrow necessitates invasive procedures to enable characterisation. Hence, although this cell population may be a key predictor of durability of antibody responses after vaccination or infection, it is rarely studied in humans^23,24^.

Most HPV vaccine immunogenicity studies evaluated only vaccine-induced serum antibody titre because breakthrough infections are barely observed in the presence of the HPV vaccine-induced antibodies. This has overshadowed evaluation of the early underlying cellular responses following HPV vaccination. Additionally, biological plausibility suggests LLPCs as the main likely source of HPV vaccine-induced antibodies while the role of Bmem and Tfh cell responses remains unclear^25,26^. Notably, almost all available studies on cellular responses following HPV vaccination used small sample sizes often reporting data obtained post-completion of multi-dose schedules^27–34^.

Booster vaccination for sub-unit vaccines plays a critical role in the reactivation of primary immune memory for enhanced long-term protection as previously reported for various conditions, including Ebola virus disease, influenza, tuberculosis, and hepatitis B^35–37^. Consequently, given the limited availability of cellular data after the first HPV vaccination dose, characterising the cell populations required for the generation and sustenance of circulating antibodies is useful, especially considering the current single-dose recommendation for the age groups primarily targeted by HPV vaccination. Evaluation of early cellular immune responses following HPV vaccination will help improve our understanding of the generation of long-term antibody-mediated protection. Such data may also give insights into what/how to leverage sub-unit vaccines for induction of durable immunity from a single vaccination dose.

Here, we evaluated cellular immunity elicited by the 9-valent HPV vaccine in Gambian females aged between 4 and 26 years, particularly focusing on plasmablasts, Bmem and Tfh cells after the first, second or third vaccination doses.

## Results

### Study groups characteristics and demographics

This study recruited a total of 120 female subjects, 40 from each of 3 age groups. Participants in each age-group were randomised into two subgroups to minimise the volume of blood drawn for evaluation of multiple cellular immune responses (Table 1).

**Table 1:** Study group characteristics

*Age group (years)*	*Number of doses*	*Group 1 Bmem analysis*	*Group 2 Plasmablast and Tfh cell analysis*
4 to 8 (median = 5)	2	*n* = 20	*n* = 20
9 to 14 (median = 10)	2	*n* = 20	*n* = 20
15 to 26 (median = 18)	3	*n* = 20	*n* = 20

**Fig. 1:**
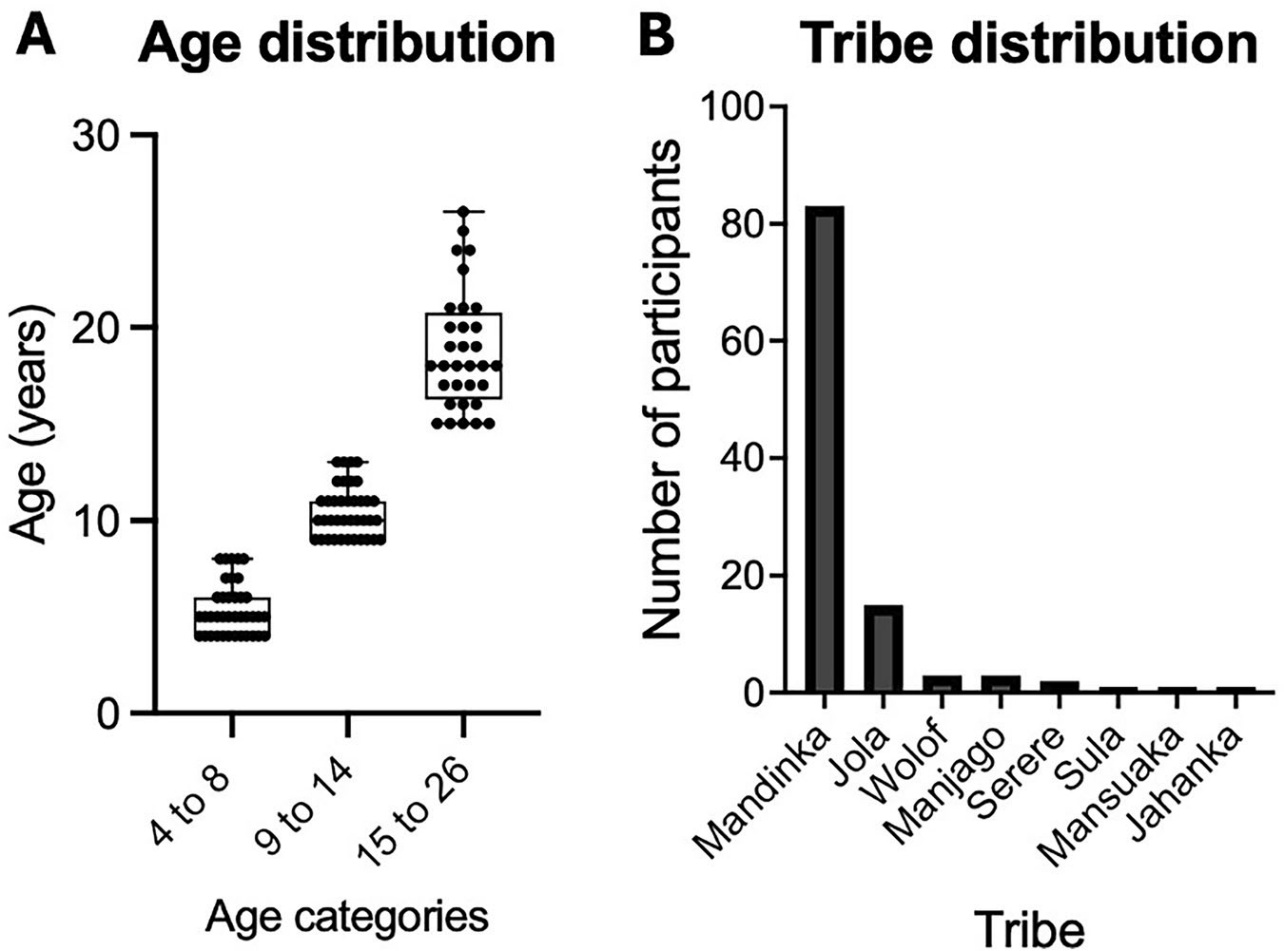
Study participants age and tribe distribution. **A** Age distribution shown in box and whisker plots with the lower, central and upper lines denoting minimum, median and maximum ages. Each dot represents an individual. **B** Tribe distribution shown in bar graphs representing the number of participants.

Age and tribe distribution are shown for participants (*n* = 108) with complete datasets for at least IgG plasmablast or IgG Bmem data across all targeted timepoints. These included 38, 38 and 32 participants aged 4-8, 9-14 and 15–26 years, respectively, who were mainly of Mandinka ethnicity (*n* = 83), followed by Jola (*n* = 15), Wolof, Manjago, Sula, Mansuaka, and Jahanka (Fig. 1).

### HPV 16/18-specific IgG and IgM plasmablast responses across vaccination doses and age groups

HPV 16/18-specific IgG and IgM plasmablasts were simultaneously enumerated across the three age groups, with data presented as pooled and age-stratified across four timepoints. Analyses were conducted for 55 participants with complete plasmablast datasets at all four evaluated timepoints. Antigen-specific plasmablast responses are expressed as the number of IgG or IgM-secreting cells per million PBMCs tested, after background subtraction.

**Fig. 2:**
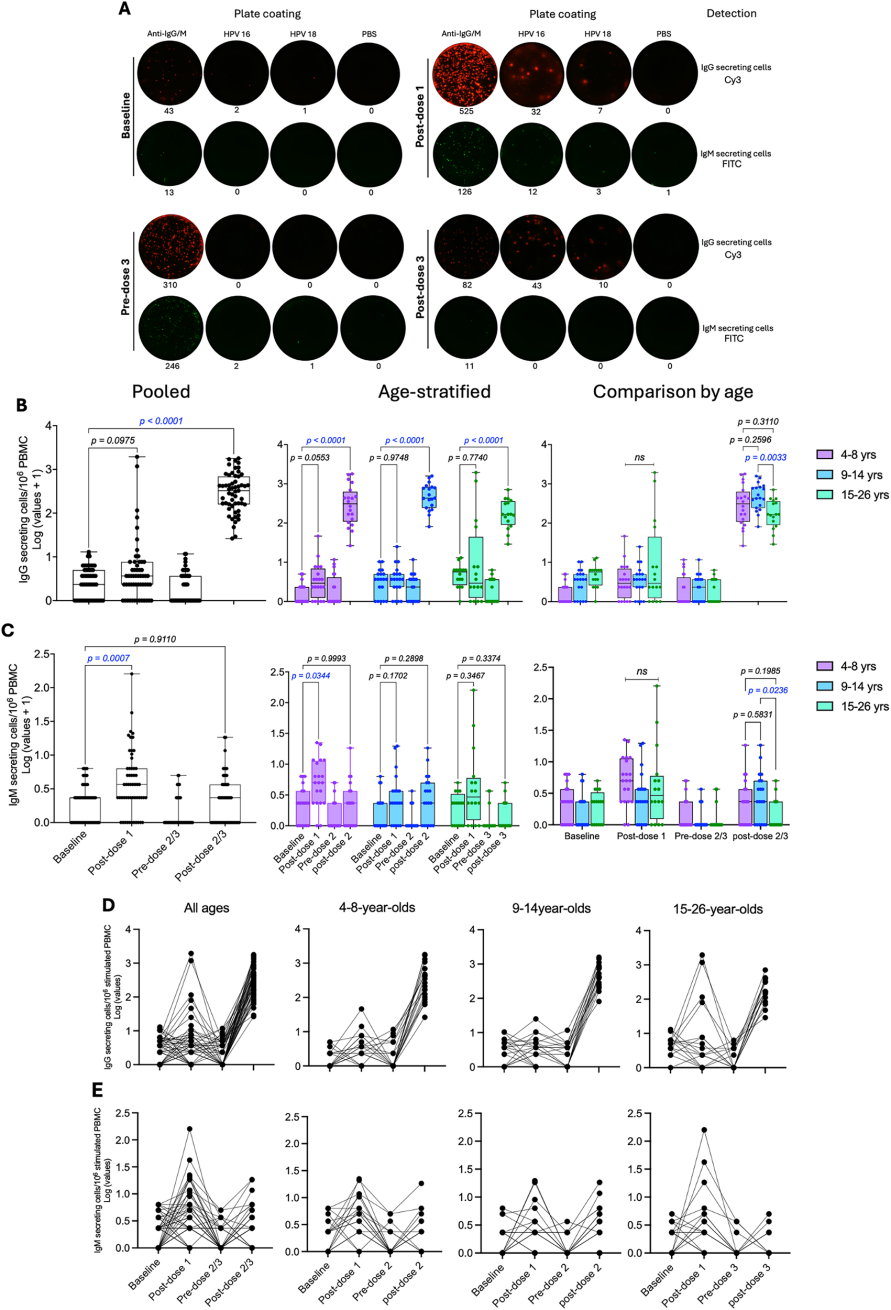
Frequencies of HPV 16/18-specific IgG and IgM plasmablast responses after Gardasil 9 vaccination. HPV 16/18-specific IgG and IgM plasmablasts were enumerated from freshly isolated peripheral blood mononuclear cells at baseline (pre-vaccination), day 7 post-dose 1, and pre- and day 7 post-dose 2 or 3 of Gardasil 9 vaccination (*n* = 20, 19, and 16 for the 4-8-, 9-14-, and 15-26-year-old groups, respectively). **A** Representative FluoroSpot readouts are shown for one participant in the 15–26-year-old group. Box and whisker plots show HPV 16-specific (**B**) IgG and (**C**) IgM secreting cells, with the lower, central, and upper lines denoting the minimum, median, and maximum values, respectively. Age groups are colour-coded, with each dot representing an individual. Statistical analyses evaluating the effects of dose number and age compared plasmablast numbers between baseline and post-dose 1, 2, or 3 timepoints. Paired one-way ANOVA with Dunnett’s adjustment and paired two-way ANOVA with Tukey’s adjustment were performed for pooled and age-stratified analyses, respectively. Statistically significant p-values (*p* < 0.05) are highlighted in blue. Line graphs show the kinetics of HPV 16-specific (**D**) IgG and (**E**) IgM secreting cells across the four evaluated timepoints. yrs—years.

**Fig. 3:**
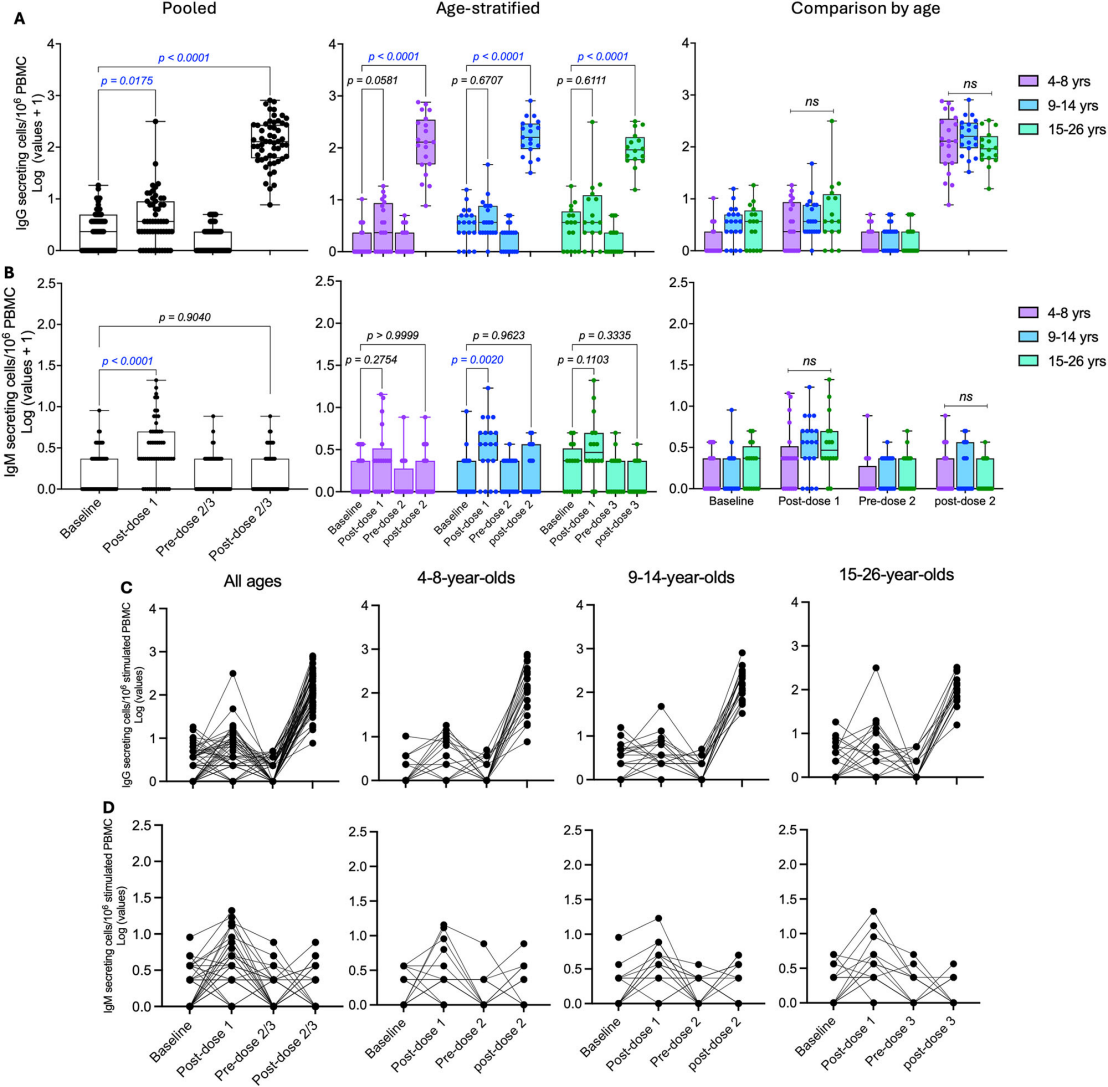
Frequencies of HPV 18-specific IgG and IgM plasmablasts responses after Gardasil 9 vaccination. HPV 18-specific IgG and IgM plasmablasts were enumerated from freshly isolated peripheral blood mononuclear cells at baseline (pre-vaccination), day 7 post-dose 1, and pre- and day 7 post-dose 2 or 3 of Gardasil 9 vaccination (*n* = 20, 19, and 16 for the 4–8-, 9-14-, and 15-26-year-old groups, respectively). Box and whisker plots show HPV 18-specific (**A**) IgG and (**B**) IgM secreting cells, with the lower, central, and upper lines denoting the minimum, median, and maximum values, respectively. Age groups are colour-coded, with each dot representing an individual. Statistical analyses evaluating the effects of dose number and age compared plasmablast numbers between baseline and post-dose 1, 2, or 3 timepoints. Paired one-way ANOVA with Dunnett’s adjustment and paired two-way ANOVA with Tukey’s adjustment were performed for pooled and age-stratified analyses, respectively. Statistically significant *p*-values (*p* < 0.05) are highlighted in blue. Line graphs show the kinetics of HPV 18-specific (**C**) IgG and (**D**) IgM secreting cells across the four evaluated timepoints. yrs—years.

Antigen-specific IgG plasmablasts were detectable in circulation 7 days after 1, 2 or 3 doses, whereas total IgG responses, used as a positive control, were detectable at all timepoints (Fig. 2A). Although a few individuals, mainly within the older age group showed high HPV 16-specific IgG plasmablast numbers after dose 1, no significant overall increase was observed in either the pooled or age-stratified analyses (Fig. 2B and Table S1). In contrast, a significant increase in HPV 18-specific IgG plasmablast numbers was observed in the pooled analysis after dose 1, again with a few older participants showing the highest responses (Fig. 3A and Table S1). Doses 2 and 3 induced markedly higher antigen-specific IgG plasmablast numbers for both HPV types (Figs. 2 and 3). ANOVA main effects analysis showed that antigen-specific IgG plasmablast numbers were significantly higher in younger participants (*p* = 0.0393 for HPV 16; *p* = 0.0218 for HPV 18). Pairwise comparisons by age revealed a significant difference in HPV 16-specific IgG plasmablast responses between two-dose recipients aged 9–14 years and three-dose recipients aged 15–26 years (Fig. 2B).

The number of antigen-specific IgM plasmablasts was generally lower than the corresponding IgG responses. The pooled analysis of HPV 16/18-specific IgM plasmablast responses revealed a significant increase after the first vaccination dose (Figs. 2C, 3B and Table S2). In the age stratified analysis, significant increases were observed in HPV 16- and HPV 18-specific IgM plasmablast numbers among the 4-8-year-olds (Fig. 2C), and 9–14-year-olds (Fig. 3B) groups respectively. Similar to the IgG plasmablast responses, post-dose 2 or 3 IgM plasmablast numbers tended to be higher in the younger age groups. Although ANOVA main effects did not reveal significant age-associated differences across the three age groups, pairwise comparisons by age indicated a significant difference in HPV 16-specific IgM plasmablast numbers between two-dose recipients aged 9-14 years and three-dose recipients aged 15–26 years (Fig. 2C).

The plasmablast kinetics across the four timepoints showed that vaccine-induced antigen-specific IgG plasmablasts showed a modest post-dose 1 increase, except for a few older individuals who exhibited elevated responses (Figs. 2D and 3C). By the pre-dose 2 or 3 timepoint, these numbers had returned to pre-vaccination levels before rising sharply following subsequent doses. In contrast, antigen-specific IgM plasmablasts exhibited the opposite trend, with the highest numbers observed after the first vaccination dose, followed by a decline at the pre-dose 2 or 3 time point and a modest increase or decrease after subsequent doses (Figs. 2E and 3D).

### HPV 16/18-specific Bmem responses across vaccination doses and age groups

**Fig. 4:**
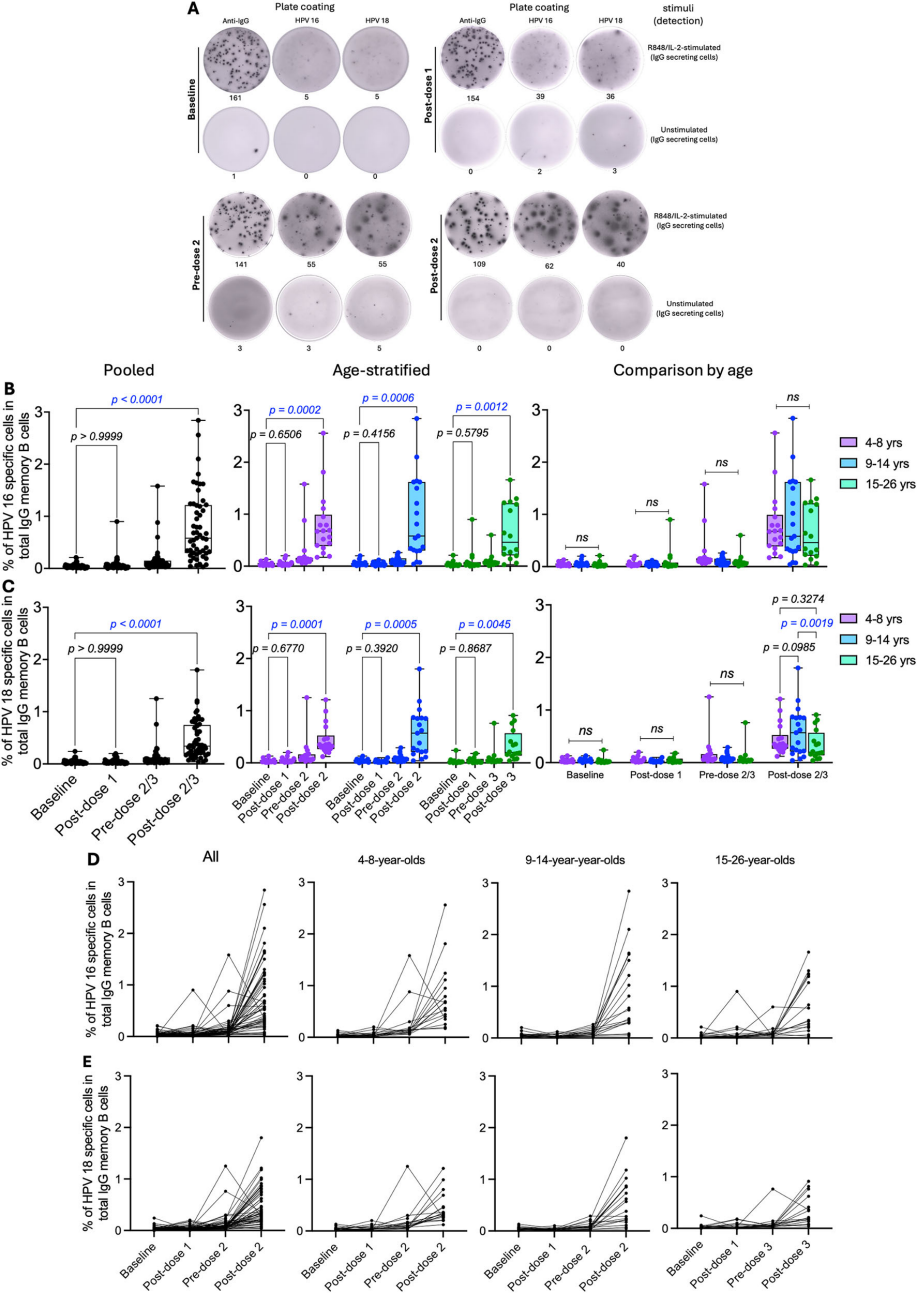
Frequencies of HPV 16/18-specific IgG Bmem responses after Gardasil 9 vaccination. HPV 16/18-specific IgG Bmem were enumerated from freshly isolated peripheral blood mononuclear cells at baseline (pre-vaccination), day 14 post-dose 1, and pre- and day 14 post-dose 2 or 3 of Gardasil 9 vaccination (*n* = 18, 19 and 17 for 4-8-, 9-14- and 15–26-year-olds, respectively). **A** Representative ELISpot readouts are shown for one participant in the 4–8-year-olds group. Box and whisker plots show (**B**) HPV 16- and (**C**) HPV 18-specific IgG Bmem responses, with the lower, central and upper lines denoting minimum, median and maximum values, respectively. Age groups are colour-coded with each dot representing an individual. Statistical tests to evaluate the effect of dose number and age compared Bmem numbers between baseline and post-dose 1, 2 or 3 timepoints. Paired One-way ANOVA with Dunnett’s adjustment and paired two-way ANOVA with Tukey adjustment were performed for pooled and age-stratified analyses, respectively. Statistically significant p values (*p* < 0.05) are highlighted in blue. Line graphs show the kinetics of (**D**) HPV 16- and (**E**) HPV 18-specific IgG secreting cells across the four evaluated timepoints. yrs – years.

HPV 16/18-specific IgG Bmem were enumerated across the three age groups, and the data are presented as pooled and age-stratified as earlier described. The analysis included 53 participants with complete datasets across all study timepoints (Fig. 4 and Table S3). Results are shown as frequencies of HPV 16/18-specific IgG Bmem within total circulating IgG Bmem after background subtraction. Antigen-specific IgG Bmem increased slightly following the first vaccination dose and rose further at subsequent timepoints, whereas total IgG Bmem, used as a positive control, was detected at high frequencies throughout (Fig. 4A). Notably, while the post-dose 1 Bmem numbers appeared high on the raw assay readouts, the corrected analysis expressing the Bmen frequencies as a proportion of total circulating IgG Bmem showed no significant increase.

No significant increases in antigen-specific Bmem frequencies were observed after the first vaccination dose in either pooled or age-stratified analyses (Fig. 4B, C). Before the administration of the second or third doses, antigen-specific Bmem frequencies showed a trend towards increase, with a robust rise following the subsequent doses (Fig. 4B, C). Although vaccine-induced Bmem responses after two or three doses tended to be higher in the younger age groups, ANOVA main effects analysis showed no overall age-associated differences. Pairwise comparisons by age revealed a significant difference in HPV 18-specific IgG Bmem frequencies between two-dose recipients aged 9–14 years and three-dose recipients aged 15–26 years (Fig. 4C). The kinetics of antigen-specific Bmem responses across all timepoints demonstrated clear increases only after the second or third dose, except for a few participants who exhibited relatively high pre-dose 2 or 3 responses that subsequently declined after vaccination (Fig. 4D, E).

### Ex vivo activated cells within the circulating Tfh pool across vaccination doses and age groups

**Fig. 5:**
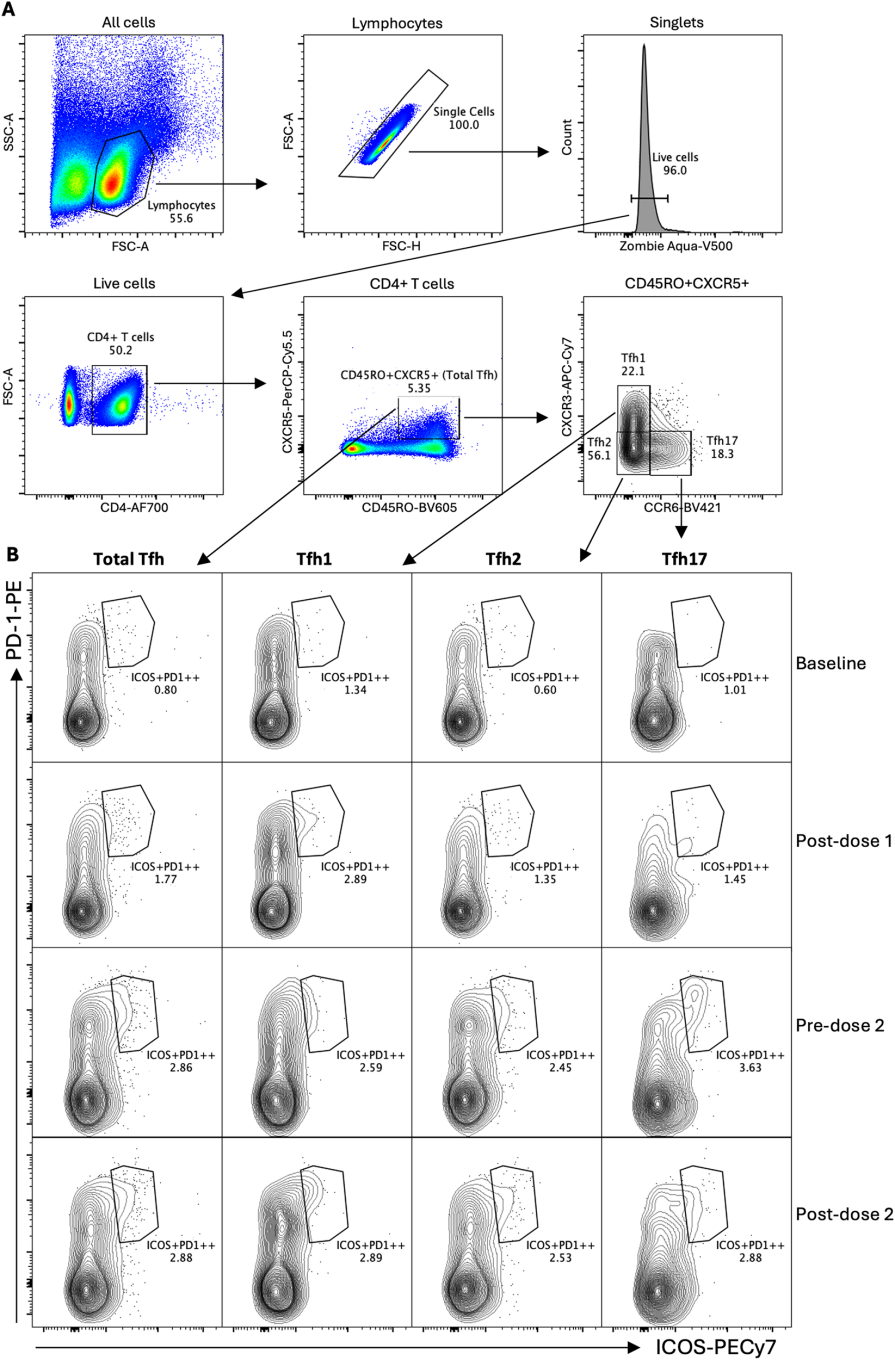
Gating strategy to identify ex vivo activated Tfh cells after Gardasil 9 vaccination. Cryopreserved PBMCs were thawed and stained using an ex vivo Tfh cell antibody panel. Data were acquired on a BD LSRFortessa III flow cytometer and analysed in FlowJo. **A** Viable, single lymphocytes were first gated from all events, and total Tfh cells were identified as CD4 + CD45RO + CXCR5+ cells. From this population, Tfh subsets were defined as Tfh1 (CXCR3 + CCR6-), Tfh2 (CXCR3-CCR6-), and Tfh17 (CXCR3-CCR6+). **B** Activated Tfh cells, defined as ICOS + PD-1++, were then identified within the total Tfh pool and each subset. Shown is a representative gating strategy from one participant in the 4–8-year-old group, consistent across all study timepoints.

Flow cytometry was used to assess Tfh cell activation within the total circulating Tfh pool following vaccination. Total Tfh cells were identified as CD4 + CD45RO + CXCR5+ cells, and the three major Tfh subsets were delineated based on CXCR3 and CCR6 expression as Tfh1 (CXCR3 + CCR6-), Tfh2 (CXCR3-CCR6-), and Tfh17 (CXCR3-CCR6+) (Fig. 5A). Activated cells within the total Tfh population and each subset were defined by co-expression of ICOS and high PD-1 levels (ICOS + PD-1++) (Fig. 5B).

**Fig. 6:**
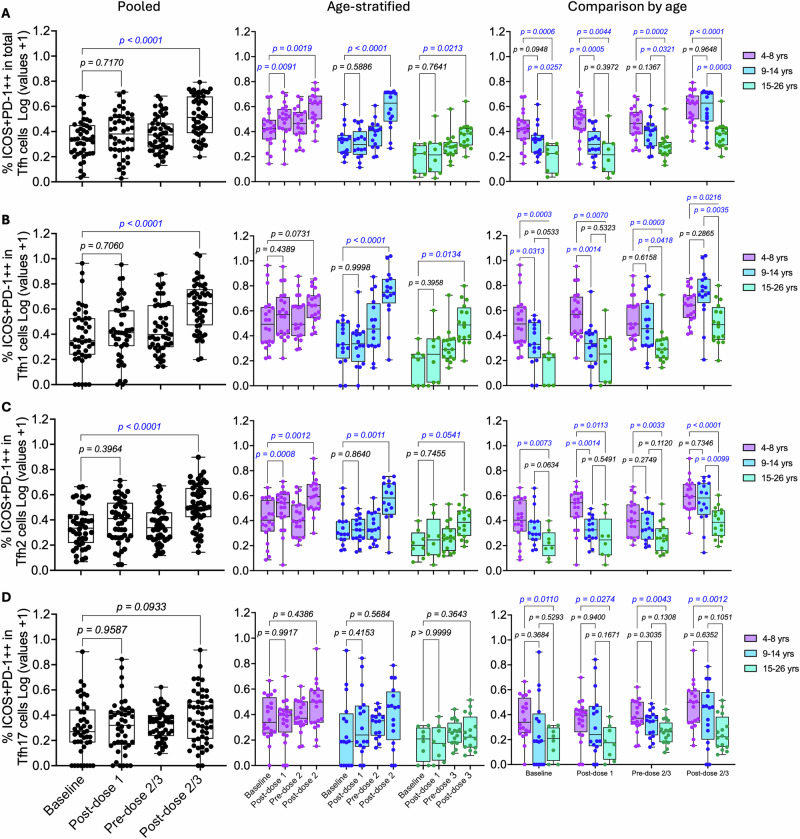
Frequencies of activated cells within the total Tfh pool after Gardasil 9 vaccination. An ex vivo flow cytometry assay was used to identify activated Tfh cells (ICOS + PD-1++) within the total circulating Tfh pool and the three Tfh subsets (Tfh1, Tfh2, and Tfh17). Box and whisker plots show frequencies of activated cells in **A** the total Tfh pool, **B** Tfh1, **C** Tfh2 and **D** Tfh17 subsets. The lower, central, and upper lines represent minimum, median, and maximum values, respectively. Age groups are colour-coded, with each dot representing an individual. Statistical analyses assessed the effects of dose number and age by comparing activated Tfh cell frequencies between baseline and post-dose 1, 2 or 3 timepoints. Unpaired one-way ANOVA with Dunnett’s adjustment and unpaired two-way ANOVA with Tukey’s adjustment were used for pooled and age-stratified analyses, respectively. Statistically significant *p* values (*p* < 0.05) are highlighted in blue. PBMC—peripheral blood mononuclear cells, yrs – years.

A robust overall activation of total Tfh cells was observed in the pooled analysis after 2 or 3 vaccine doses, with activated cells comprising mainly the Tfh1 and Tfh2 subsets (Fig. 6A-C and Table S4). A trend towards increased Tfh activation was also noted following dose 1, although this did not reach statistical significance. In age-stratified analyses, a significant increase in total Tfh cell activation was observed after dose 1 only in the 4–8-year-olds, while 2 or 3 doses induced strong Tfh activation across all age groups (Fig. 6A and Table S4).

Profiles of Tfh subset activation varied slightly by age group. Significant activation of Tfh2 cells was observed in the 4–8-year-olds after doses 1 and 2, whereas significant activation of both Tfh1 and Tfh2 subsets occurred in the two older age groups following 2 or 3 doses (Fig. 6B, C and Table S4). A consistent but non-significant increase in Tfh17 activation was observed in the two younger age groups after doses 1 and 2, while in the 15–26-year-olds, a decrease in Tfh17 activation was noted following doses 1 and 3 (Fig. 6D and Table S4).

ANOVA main effects analyses showed age and dose-associated differences in the frequencies of activated ICOS + PD-1 + + Tfh cells (*p* = 0.00 l). Pairwise comparisons by age showed a clear trend, with activated Tfh cell frequencies being highest in the 4–8-year-olds, intermediate in the 9–14-year-olds and lowest in the 15–26-year-olds. This trend was consistent across timepoints, except for Tfh1 post-dose 2 or 3, where activation was highest in the 9–14-year-olds (Fig. 6B), and Tfh17 at baseline, where activation in the 15–26-year-olds was slightly higher than in the 9–14-year-olds (Fig. 6D).

### HPV 16/18-specific Tfh responses across vaccination doses and age groups

**Fig. 7:**
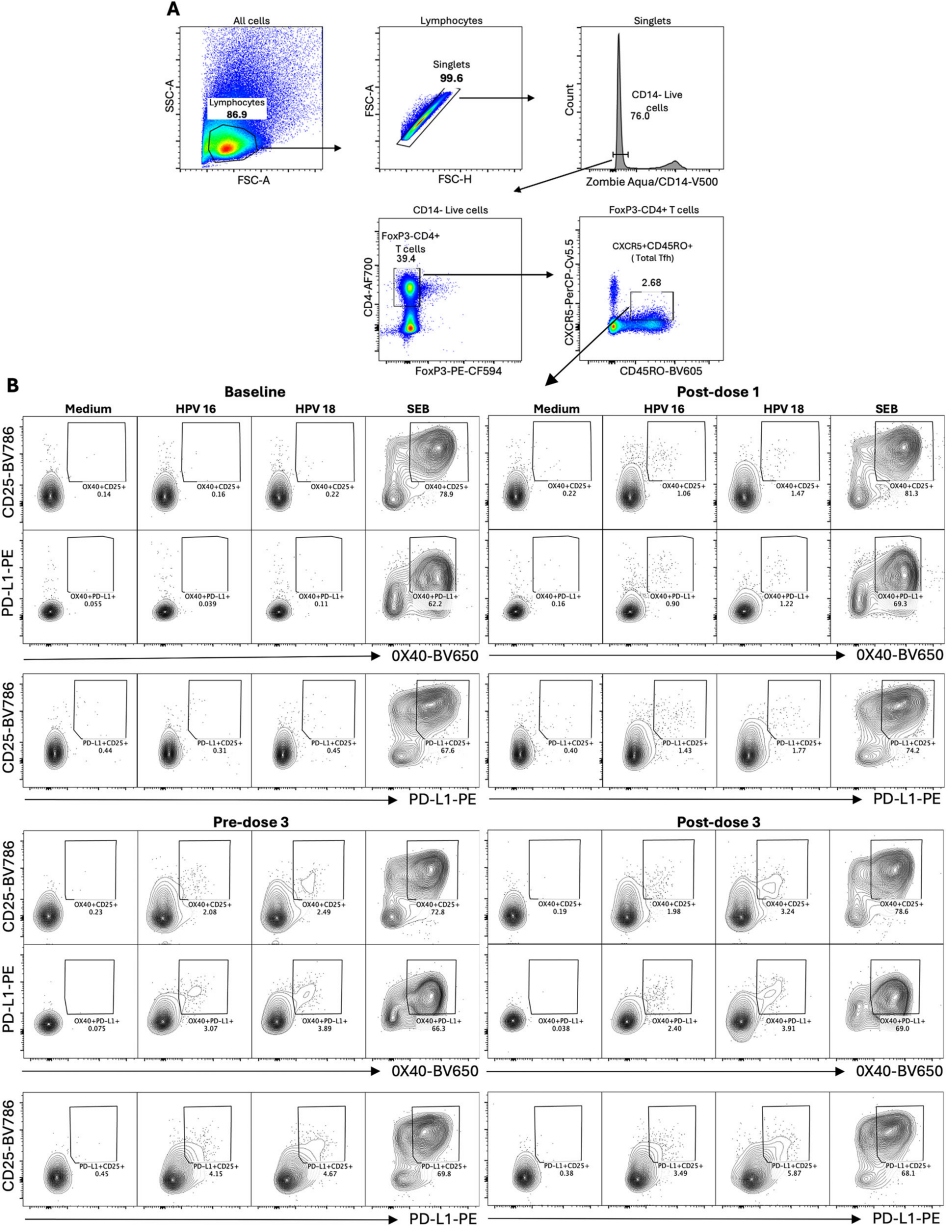
Gating strategy to identify activated HPV 16/18-specific Tfh cells after Gardasil 9 vaccination. Frozen PBMCs were thawed and pre-stimulated in culture for 18 h with HPV 16 or HPV 18 VLPs, a mitogen (SEB) positive control, or cultured in medium only without stimulation (Medium). Stimulated cells were stained with the AIM antibody panel, and data were acquired on BD LSRFortessa III flow cytometer with gating performed in FlowJo. **A** Lymphocytes were first gated from all cells, followed by exclusion of dead cells and CD14+ monocytes. Total Tfh cells were then identified as FoxP3-CD4 + CD45RO + CXCR5+. **B** Activated cells were identified using AIM markers as OX40 + CD25 + , OX40 + PD-L1+ and PD-L1 + CD25+ across all stimulation conditions. Shown is a representative gating strategy from one subject in the 15–26-year-old group. VLPs - virus-like particles; SEB staphylococcal enterotoxin B; AIM activation-induced marker.

To evaluate antigen-specific Tfh cell activation following vaccination, an activation-induced marker (AIM) flow cytometry assay was performed. PBMCs were cultured for 18 h with vaccine antigens (HPV 16 or HPV 18 VLPs), a mitogen positive control (staphylococcal enterotoxin B, SEB), or without stimulation to assess background activation. The same markers used for ex vivo identification of total Tfh cells were included, with the addition of FoxP3 to exclude regulatory T cells that can upregulate CXCR5 expression during culture re-stimulation. Tfh cell subsets were not evaluated in the AIM panel due to the expected low frequencies of antigen-specific cells, which would make further subdivision unreliable. Total Tfh cells in the AIM panel were therefore defined as FoxP3-CD4 + CD45RO + CXCR5+ (Fig. 7A). Within this population, activated Tfh cells were identified under each stimulation condition based on co-expression of three activation markers: OX40 + CD25+, OX40 + PD-L1+, and PD-L1 + CD25+ (Fig. 7B).

**Fig. 8:**
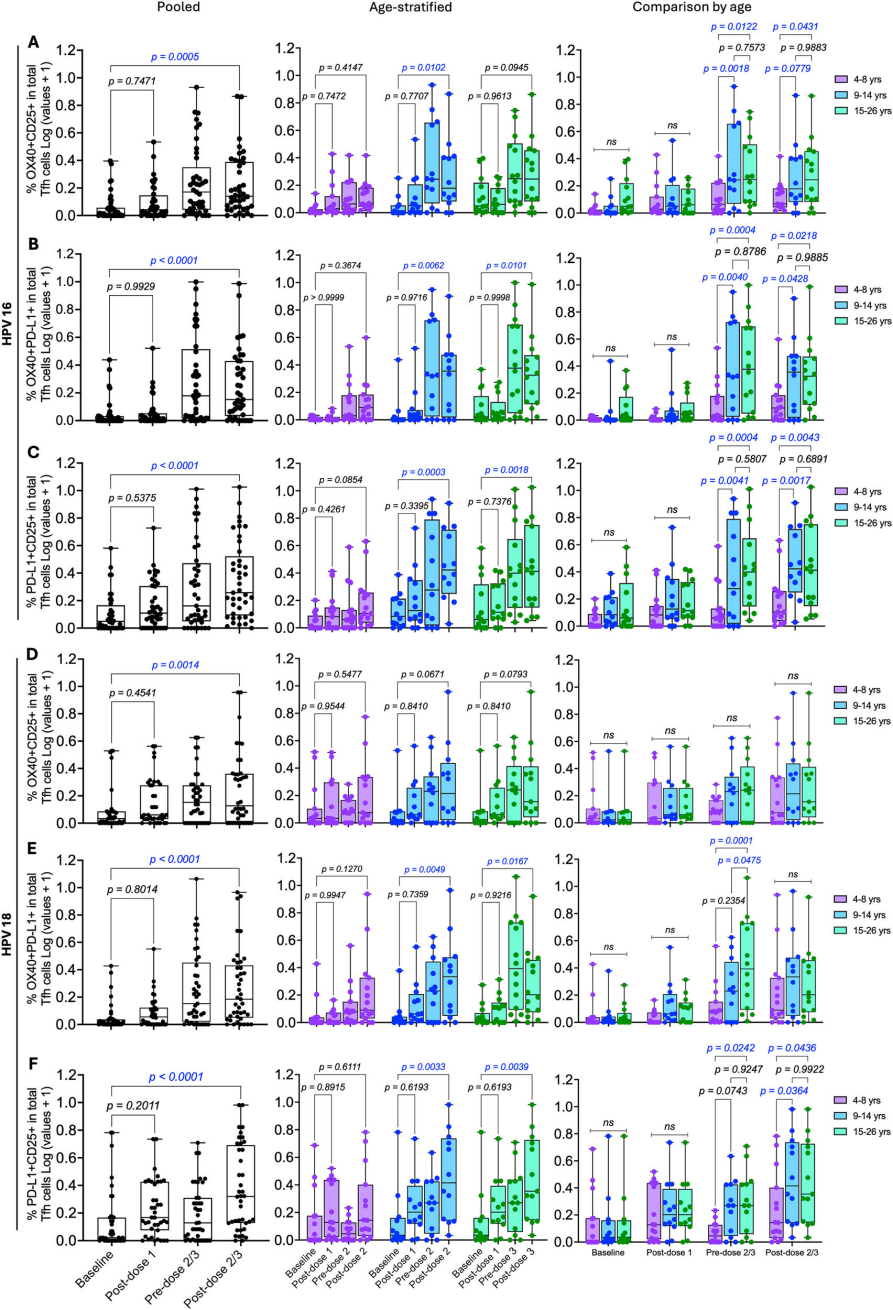
Frequencies of activated HPV 16/18-specific Tfh cells after Gardasil 9 vaccination. Activated HPV 16/18-specific Tfh cells were identified using an AIM flow cytometry assay with the phenotypes OX40 + CD25+, OX40 + PD-L1+ and PD-L1 + CD25+ within the total circulating Tfh cell pool. Box and whisker plots show activated HPV 16-specific Tfh cells based on **A** OX40 + CD25+, **B** OX40 + PD-L1+ and **C** PD-L1 + CD25+, and HPV 18-specific Tfh cells based on **D** OX40 + CD25+, **E** OX40 + PD-L1+ and **F** PD-L1 + CD25+. The lower, central, and upper lines indicate the minimum, median, and maximum values, respectively. Age groups are colour-coded, with each dot representing an individual. Statistical evaluation of the effects of dose number and age compared activated Tfh cell frequencies between baseline and post-dose 1, 2, or 3 timepoints. Unpaired one-way ANOVA with Dunnett’s adjustment and unpaired two-way ANOVA with Tukey adjustment were performed for pooled and age-stratified analyses, respectively. Statistically significant *p* values (*p* < 0.05) are highlighted in blue. VLPs virus-like particles, SEB staphylococcal enterotoxin B, AIM activation-induced marker.

Overall, comparable signal detection was observed across the three AIM marker combinations. In the pooled analysis, there was a trend toward increased frequencies of antigen-specific activated Tfh cells for both HPV types across all AIM combinations, although this did not reach statistical significance (Fig. 8A-F and Tables S5, S6). Activation increased to levels significantly above baseline following dose 2 or 3. Upon age stratification, a modest increase in activated Tfh cell frequencies was observed in all age groups post-dose 1. After doses 2 or 3, the OX40 + PD-L1+ and PD-L1 + CD25 + AIM combinations identified significant induction of antigen-specific cells in the two older age groups for both HPV types, whereas a significant increase in OX40 + CD25+ frequencies was observed in 9–14-year-olds for HPV 16 only. In 4–8-year-olds, post-dose 1 and 2 antigen-specific Tfh cell frequencies tended to be higher than baseline but did not reach statistical significance. Notably, although the statistical analysis of dose number effects across all characterised cell populations compared responses at baseline with those post-doses 1, 2 or 3 to minimise multiplicity errors, in some instances, activated Tfh cell frequencies at the pre-dose 2 or 3 timepoint were similar to, or even higher than, those detected post-dose 2 or 3.

ANOVA main effects revealed a significant age effect on AIM Tfh cell activation: for HPV 16, OX40 + CD25 + , *p* = 0.0008; OX40 + PD-L1 + , p < 0.0001; PD-L1 + CD25 + , *p* < 0.0001; and for HPV 18, OX40 + PD-L1 + , *p* = 0.0098; PD-L1 + CD25 + , *p* = 0.0081. Follow-up pairwise comparisons by age showed higher cell activation in the older age groups (Fig. 8A–F), contrasting the trend observed in Tfh ex vivo activation.

## Discussion

This study detected higher overall numbers of IgG than IgM plasmablasts following HPV VLP vaccination. This finding is in line with the well-demonstrated induction of predominantly protective IgG antibodies not only by HPV but also by other vaccines, including tetanus, *Haemophilus influenzae* type b, pneumococcus and yellow fever^38–40^. The first vaccination dose induces lower and delayed plasmablast numbers mainly from activation of naïve B cells compared to subsequent doses, which induce rapid reactivation of Bmem to differentiate into antibody-secreting cells^41^. The plasmablast numbers detected in this study followed this trend. Additionally, the detection of the highest post-dose 1 IgG plasmablasts in the oldest age group may indicate prior natural exposure to HPV. Contextualising this to the dynamics of documented antibody titre following HPV vaccination^5,11–13^, a single HPV vaccination dose induces IgG antibody titres that are significantly lower than those measured after two or three doses, a similar pattern to the plasmablast responses observed in this study. Importantly, antibody titres from single or multiple vaccination doses are similarly sustained without waning. A recent post hoc analysis of five Gardasil 9 trials reported robust immunogenicity against a range of baseline characteristics in men despite some variability between studies or populations^14^. At 10 years post-vaccination, HPV 16/18 neutralising antibodies from a single dose of the Gardasil vaccine were reported to be about two times higher than those induced by natural infection^6^ although these results are contrasted by findings from a recent post hoc analysis of Gardasil 9 responses^14^. This may suggest that despite the low numbers of IgG plasmablasts detected in circulation after the first Gardasil 9 vaccination dose in our current study, a single dose may be able to generate sufficient LLPCs in the secondary lymphoid organs to sustain the observed long-term antibody titres. The use of blood samples in this study meant LLPCs could not be characterised since after their generation in the germinal centres, they home to special niches in the bone marrow and do not recirculate^42^.

The low frequencies of Bmem detected following the first vaccination dose and increasing after subsequent doses are in line with the expectation that, as observed in plasmablast responses, overall immune activation was lower than after subsequent doses. Bmem reactivation by dose 2 or 3 causes them to proliferate and differentiate, increasing the number of the Bmem themselves as well as plasmablasts^43,44^. In contrast to plasmablasts, Bmem recirculate and are detectable in blood for years following infection or vaccination^45^. Although we investigated short-term immune responses after Gardasil 9 vaccination, previous studies reported HPV-specific Bmem in circulation at 4–6 years after both Cervarix and Gardasil vaccination, and more recently up to 10 years following Gardasil vaccination^29,34^. Notably, high individual-level titres of vaccine-specific Bmem detected in circulation before second or third vaccination doses have been reported to inversely correlate with subsequent booster dose responses. Indeed, the absence of an increase in Bmem responses post-dose 2 or 3 in few subjects who had these responses at high levels pre-dose 2 or 3 aligns with findings from a recent report of long-term Bmem responses following 2 or 3 doses of the Gardasil vaccine^34^. This may be due to high antibody titre from earlier vaccination doses suggested to obscure the vaccine antigen in subsequent doses given after short intervals, thereby preventing Bmem reactivation^28^. The unexpected decline in Bmem numbers following dose 2 or 3 in a few individuals may have been by chance or perhaps due to biological variations in individual responses to vaccination.

Several proposals have been put forward to support LLPCs as the most likely source of long-term antibodies induced by HPV vaccination. Although there is no established correlate of protection against HPV, the vaccine-induced antibodies maintained significantly above levels induced by natural infection have been suggested to protect against the expected low doses of transmissible virus^46^. Additionally, the absence of blips in the profiles of antibody titres after they attain a plateau phase following vaccination is thought to indicate little or no contribution of Bmem in maintaining the long-term antibody protection^25^. This possibility is further supported by the absence of a direct positive correlation between HPV-specific Bmem and antibody titres after vaccination^29^. However, given the complex and coordinated nature of the immune system, the relative contributions of both Bmem and LLPCs in generation of long-term antibodies needs to be considered especially, when evaluating the immunogenicity of a single-dose vaccination schedule of a subunit vaccine expected to induce limited immune activation.

Considering the age-dependent dynamics of HPV infection and that the vaccines are primarily recommended for administration before sexual debut, it is possible that the role of Bmem in sustaining long-term circulating antibodies for protection against incident HPV infections may be realised in future longer-term studies. Indeed, a 10-year follow up after Gardasil vaccination reported a decrease in plateau-phase antibody titres from the single dose group to similar levels induced by natural infection in unvaccinated individuals^8^. Additionally, increased antibody titre observed at the time the vaccinees were married was suggested to be from Bmem reactivation due to possible increased exposure to HPV^8^. Antibody response kinetics differ between HPV types. The proportion of individuals with detectable neutralising antibodies was lower for HPV 18 (49.1%) compared to HPV 16 (97.8%)^8^. A recent single-dose randomised trial of the Gardasil 9 and Cervarix vaccines reported that single-dose non-inferiority to 2 and 3 doses for HPV 18 seropositivity was not met at 5 years following vaccination, hence the impact this may have on long-term single dose protection needs to be investigated^12^. In contrast to the stated seropositivity levels, the efficacy of Gardasil 9 for HPV 18 between 4 and 5 years after vaccination in ESCUDDO trial was the same for both one dose and two dose schedules (97.1% and 97.6%)^19^.

Given the requirement of Tfh cell help for B cells to differentiate into Bmem and LLPCs, the observed similar kinetics of plasmablast, Bmem and Tfh cell responses was relevant to the coordinated role played by these cell populations in antibody generation. The induction of lower AIM-based Tfh cell response in the youngest age group, unlike all other evaluated cell responses was surprising and remains to be understood. Nevertheless, the Tfh cell activation increased by number of doses across all age groups similar to the B cell responses, further supporting their possible contribution to antibody responses. Although Tfh cell responses are expected to decline with age due to immunosenescence, there is limited understanding on their dynamics between the age groups targeted in this study. Additionally, the AIM assay has neither previously been used to identify HPV-specific Tfh responses, nor has it been used to compare Tfh responses to other antigens in different age groups which may possibly explain if the performance of the AIM markers used is affected by age.

The increased activation total Tfh cells, particularly the Tfh1 and Tfh2 subsets, which are critical for maintenance of long-term antibodies, indicate their potential contribution to subsequent antibody production following HPV vaccination. We note the ex vivo-characterised Tfh responses may have been confounded by bystander activation, since the PD-1 and ICOS markers are not strictly antigen specific.

In terms of the age-associated differences in the activation of characterised cell responses, the higher B cell responses observed in younger age-groups is consistent with results from studies evaluating antibody responses that consistently report higher titre in adolescents than adults vaccinated against HPV^47^.

While Bmem and Tfh cells are required to boost antibody responses following reactivation by secondary antigen encounter, plasmablasts have a direct role as antibody producers. Such differences in addition to variations in the timing of maturation and localisation of these cell populations may contribute to different dynamics of their maintenance as previously reported^29,30^. It is therefore not surprising that our results did not show consistent profiles of the B cell and Tfh cell responses at some timepoints.

We note some limitations of this study. By measuring cellular immune responses to multiple vaccination doses at similar timepoints, peak responses from the first dose may have been missed, as may be delayed compared to responses to subsequent doses. Future similar studies with sufficient sample size could initially optimise for target timepoints for detection of peak responses. The measurement of different cellular responses in different subgroups did not allow for correlation analyses for example between Bmem and Tfh responses. This may be a common challenge in studies evaluating multiple parameters where further subject subgrouping is necessary to minimise the blood volume drawn from each participant. Although the numbers of circulating vaccine-induced plasmablasts may give useful insights on the extent to which HPV vaccines induce plasma cell response, they may not truly represent bone-marrow LLPCs. Similarly, Bmem and Tfh responses from peripheral circulation may not truly represent the germinal centre responses. The corresponding serology results to the cellular results presented in this study are not presented which would have allowed for controlling for prior infections at baseline and correlational analyses at subsequent timepoints. However, the trial serology data is being analysed separately after which we will be able to perform the correlational analyses with the cellular data for participants in this study.

Despite these limitations, our results provide valuable evidence of the dynamics and magnitude of HPV vaccine-induced B cell and Tfh responses in peripheral blood. Correlating these cellular responses to the corresponding antibody levels in the future will provide more useful insights into systemic correlates of the vaccine immunogenicity across age groups. This study characterised cellular responses to HPV 16/18 that contribute the highest global burden of cervical cancer. Given the variable antibody responses observed across HPV types, future characterisation of responses to the other seven HPV types contained in the study vaccine may provide useful data^48^. We characterised responses in female participants for feasibility reasons. Previous studies reported similar antibody responses at about one month to eight years following Gardasil 9 vaccination of boys and girls aged 9 to 15 years^49,50^ while a recent post hoc analysis of five Gardasil 9 trials showed differences in antibody responses between males and females^14^. It may be of interest to understand how cellular responses compare between males and females.

Taken together, the integrated evaluation of both B cell and Tfh cell responses in this work provide a broader understanding of adaptive cellular immunity induced by HPV vaccination. Our findings add to the existing literature demonstrating higher immunogenicity of HPV vaccines in young age groups and longer vaccination dose intervals.

The head-to-head comparison of HPV vaccine-induced immunogenicity across the three evaluated age groups in this work has not been previously reported. Consequently, the finding that the vaccine induced higher cellular responses in younger participants, including children aged 4–8 years, in whom HPV vaccination has not been tested before, warrants further investigation. Considering the low Bmem numbers observed at 12 months post-dose 1, low-responders and immunocompromised individuals may be at risk of being infected and developing disease in case their vaccine-induced antibody titre wanes to levels warranting recruitment of Bmem following infection, hence the need for continued monitoring of immunogenicity from single-dose HPV vaccination.

## Methods

### Study design

This study was nested within a randomised non-inferiority trial (NCT03832049) of the 9-valent HPV vaccine in The Gambia. The trial recruited a total of 1720 female participants across three age groups: 4–8- and 9–14-year-olds, who received one or two vaccination doses and 15–26-year-olds, who received three vaccination doses. Within the trial, the last 60 participants to be recruited in each age group were selected to form this sub-study. Informed consent was obtained from adult study participants or from parents of participants aged below 18 years. This study was conducted according to the ethical principles of the Declaration of Helsinki. The protocol was reviewed and approved by The Gambian Government and Medical Research Council Unit the Gambia Joint Ethics Committee (GG/MRCG JEC Ref: SCC 1597v2.1) as well as the London School of Hygiene and Tropical Medicine Research Ethics Committee (LSHTM Ethics Ref: 16076). The study also received regulatory approval from the Medicines Control Agency of the Government of The Gambia.

### Blood sampling and isolation of PBMCs

Study subjects were grouped into three based on age and vaccination schedule: Group 1 and 2, 4–8- and 9–14-year-olds who received two vaccination doses (0, 12 months), and Group 3, 15-26-year-olds who received three vaccination doses (0, 2, and 12 months). Blood samples were collected from all participants immediately before any vaccination and at either day 7 or 14 after the first, second (groups 1 and 2) and third (group 3) vaccination doses (Fig. S1). Sampling timepoints were informed by previous studies that characterised cellular immune responses after HPV vaccination.^30,32^ Collected samples were transported to the nearest MRC Gambia research laboratories in Keneba field station for processing within a maximum of 6 h after collection. PBMCs were isolated at room temperature by density gradient centrifugation in Lymphoprep Density Gradient Medium (Axis-Shield Limited, Scotland), protected from light. The freshly isolated PBMCs were subsequently analysed by FluoroSpot for IgG and IgM plasmablast responses pre- and day 7 post-vaccination or by ELISpot for IgG Bmem responses pre- and day 14 post-vaccination. Some PBMCs from the samples analysed for plasmablast responses were viably cryopreserved in freezing medium (10% Dimethyl sulfoxide in FBS) at −80 ^o^C for later testing of Tfh cell responses by flow cytometry within 12 months of cryopreservation. For all the cellular responses analysed, we did not control for prior infection because seronegative individuals were not identified at baseline. Potential responses from prior infection especially in the older age groups may confound post-dose 1 vaccine responses.

### ELISpot and FluoroSpot

ELISpot and FluoroSpot procedures were performed using commercial kits containing all reagents for the two assays (Mabtech AB, Stockholm, Sweden) following the manufacturer’s instructions. IgG-secreting Bmem were quantified using ELISpot. Briefly, freshly isolated PBMCs were pre-cultured for 6 days with mitogenic stimulation using the Toll-like receptor 7/8 agonist (R848) and recombinant anti-human IL-2, or without stimulation. Ninety-six-well plates were coated with HPV 16/18 VLPs (Merck Sharp and Dohme Corp., USA) at 4 °C overnight, washed and incubated with pre-stimulated PBMC for 20 h. During incubation, HPV 16/18-specific IgG antibodies secreted by B cells were captured by the HPV VLPs and the cells were removed by washing in PBS. The captured antigen-specific antibodies were then detected using a secondary biotinylated antibody, streptavidin-ALP conjugate and ALP substrate (Nitro Blue Tetrazolium/5-bromo-4-chloro-3-indolyl phosphate -NBT/BCIP).

Both IgG- and IgM-secreting plasmablasts were simultaneously enumerated from freshly isolated PBMCs using FluoroSpot. Plate coating and cell incubation steps were performed as described for ELISpot. Simultaneous detection of IgG- and IgM-secreting cells was performed using fluorescently labelled secondary antibodies.

Following the laboratory analysis of freshly isolated PBMCs for PC and MBC responses at the Keneba field station, all FluoroSpot and ELISpot plates were transported to the Fajara main laboratory at room temperature, protected from light to maintain the integrity of the spots and the plates were read within 4 days from the day they were developed.

ELISpot and FluoroSpot spots were counted using an AID ELISpot reader (AID Autoimmun Diagnostika GmbH, Straßberg, Germany) located at the MRC Gambia, Fajara immunology laboratory.

### Flow cytometry

Frozen PBMCs were transported in dry ice from the Keneba field station to the main MRC Gambia Fajara biobank from where they were analysed for the frequency and phenotype of activated Tfh cells both ex vivo and after 18-h stimulation using the AIM antibody panel. All flow cytometry experiments from staining of cells to data acquisition were performed at the MRC Gambia, Fajara immunology laboratory. Concentrations of all reagents were pre-optimised and appropriate controls were used. Briefly, on the day of analysis, PBMCs were moved from the biobank to the laboratory in dry ice, thawed immediately in a water bath at 37 °C, washed in complete cell culture medium with centrifugation and separated into two aliquots (for ex vivo and AIM analysis). For ex vivo analysis, thawed cells were first temperature-acclimatised by incubating at 37 °C and 5% CO₂ for 1 h before staining. Cells to be analysed using the AIM assay were stimulated in culture with either HPV 16 or 18 for antigen-specific response, with a mitogenic positive control (SEB; Sigma Aldrich, Saint Louis, USA), or cultured without stimulation to identify background activation. Surface staining for both panels was similar, except for the inclusion of the AIM and FoXP3 markers in the AIM panel. Cells were first stained for viability using Zombie Aqua dye-V500 (BD Biosciences, New Jersey, USA) before washing and staining with the antibody cocktail for all surface markers. After surface staining, intracellular staining was performed for FoxP3 in the AIM panel. Details of all antibodies and dyes used in both panels are shown in Tables S7 and S8. Data were acquired on the same day of staining using BD LSRFortessa III flow cytometry located at MRC Gambia, Fajara immunology laboratory and FACs Diva software and exported for gating in FlowJo Software for Mac v10.10.0 (BD Life Sciences). A minimum of 2000 gated total Tfh events were acquired for both ex vivo and AIM assays. The viability of all cell samples analysed by flow cytometry ranged between 75 and 90% and was consistent across batches.

### Statistical analysis

ELISpot, FluoroSpot and flow cytometry data were collated in CSV format and analysed using GraphPad Prism v10.2.2 for Mac. Non-normal data were log-transformed before analysis. Pooled analysis (all three age groups) was performed to evaluate the overall effect of vaccination dose number using one-way ANOVA with Dunnett’s adjustment for multiple comparisons. Age-stratified analysis was performed to evaluate the effects of age and dose number using two-way ANOVA with Tukey adjustment for multiple comparisons. To further minimise errors from multiple comparisons, statistical comparisons for evaluation of dose number effects were performed between baseline and post-dose 1, 2 or 3, depending on group vaccination schedules.

## Supplementary information

Supplementary Information

## Data Availability

All data supporting the findings of this study are contained within the article and its supplementary content. The raw data can be obtained from the corresponding author upon request.
