# Supplementary material for: Plasmablast, memory B cell and T follicular helper cell responses after human papillomavirus vaccination: effect of dose number and age
Source: NPJ Vaccines. 2026 Feb 21;11:77. doi: 10.1038/s41541-026-01408-w (PMC13035850; doi:10.1038/s41541-026-01408-w)
Supplement: Supplementary file 1 — Supplementary Information [file 41541_2026_1408_MOESM1_ESM.pdf]

Supplementary information

Plasmablast, memory B cell and T follicular helper cell responses after human papillomavirus vaccination: effect of dose number and age

Eunice W. Kiamba[1,3]*, Dolapo O. Ajiboye[1,3], Adedapo Olufemi Bashorun[1,3], Mamie Ndeban Jallow[1], Lamin Drammeh[5], Samba Bah[1], Tijan Jobarteh[1], Francis Kanu[1], Ousubie Jawla[1], Jobarteh Lamin[1,3], Anne Segonds-Pichon[1,3], Martin J Holland[1,3], Martin R. Goodier[1,4], Sophie Roetynck[2,3], Ed Clarke[1,3]

Affiliations:

[1]Vaccines and Immunity Theme, MRC Unit The Gambia at London School of Hygiene and Tropical Medicine, P.O. Box 273, Banjul, The Gambia.

[2]Disease Control and Elimination Theme, MRC Unit The Gambia at London School of Hygiene and Tropical Medicine, P.O. Box 273, Banjul, The Gambia.

[3]Department of Clinical Research, London School of Hygiene and Tropical Medicine; Keppel Street, London WC1E 7HT, United Kingdom.

[4]Department of Infection Biology, London School of Hygiene and Tropical Medicine; Keppel Street, London WC1E 7HT, United Kingdom.

[5]Edward Francis Small Teaching Hospital, FC4C+WW4, Banjul, The Gambia.

*Corresponding author. Eunice.kiamba@lshtm.ac.uk

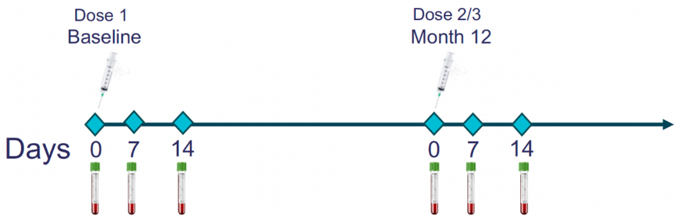

Fig. S1. Vaccination and blood sampling timepoints

For the samples used in this study, females aged between 4 and 14 years received 2 vaccination doses (12 months interval) while those aged between 15 and 26 years received 3 doses (2, 10 months intervals). Blood samples were collected immediately before vaccination (day 0) and at either day 7 or 14 after vaccination, as shown at baseline and at 12 months post-dose 1. The day 7 samples were used to evaluate plasmablast and T follicular helper cell responses while day 14 samples were tested for memory B cell responses.

Table S1. Number of HPV 16/HPV 18-specific IgG secreting cells per 10^6 PBMCs

A: HPV 16 pooled

	**All ages**	**4 to 26 years; n = 55**			
	Sampling timepoint	Baseline	Post-dose 1	Pre-dose 2/3	Post-dose 2/3
No. of IgG secreting cells per 10^6 PBMC	25% Percentile	0	1	0	157
	Median	1	3	0	325
	75% Percentile	4	7	3	691

B: HPV 18 pooled

	**All ages**	**4 to 26 years; n = 55**			
	Sampling timepoint	Baseline	Post-dose 1	Pre-dose 2/3	Post-dose 2/3
No. of IgG secreting cells per 10^6 PBMC	25% Percentile	0	1	0	60
	Median	1	3	0	127
	75% Percentile	4	8	1	271

C: HPV 16 age-stratified

	**Age stratified**	**4 to 8 years; n = 20**				**9 to 14 years; n = 19**				**15 to 26 years; n = 16**			
	Sampling timepoint	Baseline	Post-dose 1	Pre-dose 2	Post-dose 2	Baseline	Post-dose 1	Pre-dose 2	Post-dose 2	Baseline	Post-dose 1	Pre-dose 3	Post-dose 3
No. of IgG secreting cells per 10^6 PBMC	25% Percentile	0	0	0	108	0	1	0	243	2	0	0	90
	Median	0	2	0	310	3	3	1	432	5	2	0	165
	75% Percentile	1	6	3	636	4	4	3	827	5	61	3	361

D: HPV 18 age-stratified

	**Age stratified**	**4 to 8 years; n = 17**				**9 to 14 years; n = 20**				**15 to 26 years; n = 18**			
	Sampling timepoint	Baseline	Post-dose 1	Pre-dose 2	Post-dose 2	Baseline	Post-dose 1	Pre-dose 2	Post-dose 2	Baseline	Post-dose 1	Pre-dose 3	Post-dose 3
No. of IgG secreting cells per 10^6 PBMC	25% Percentile	0	0	0	47	1	1	0	93	0	1	0	57
	Median	0	1	0	128	3	3	1	159	3	3	0	91
	75% Percentile	1	8	1	347	4	7	1	292	5	11	1	160

Table S2. Number of HPV 16/HPV 18 specific IgM secreting cells per 10^6 PBMCs

A: HPV 16 pooled

	**All ages**	**4 to 26 years; n = 55**			
	Sampling timepoint	Baseline	Post-dose 1	Pre-dose 2/3	Post-dose 2/3
No. of IgM secreting cells per 10^6 PBMC	25% Percentile	0	1	0	0
	Median	1	3	0	1
	75% Percentile	1	5	0	3

B: HPV 18 pooled

	**All ages**	**4 to 26 years; n = 55**			
	Sampling timepoint	Baseline	Post-dose 1	Pre-dose 2/3	Post-dose 2/3
No. of IgM secreting cells per 10^6 PBMC	25% Percentile	0	1	0	0
	Median	0	1	0	0
	75% Percentile	1	4	1	1

C: HPV 16 age stratified

	**Age stratified**	**4 to 8 years; n = 17**				**9 to 14 years; n = 20**				**15 to 26 years; n = 18**			
	Sampling timepoint	Baseline	Post-dose 1	Pre-dose 2	Post-dose 2	Baseline	Post-dose 1	Pre-dose 2	Post-dose 2	Baseline	Post-dose 1	Pre-dose 3	Post-dose 3
No. of IgM secreting cells per 10^6 PBMC	25% Percentile	0	1	0	0	0	0	0	0	0	0	0	0
	Median	1	4	0	1	0	1	0	1	1	2	0	0
	75% Percentile	3	10	1	3	1	3	0	4	2	5	0	1

D: HPV 18 age-stratified

	**Age stratified**	**4 to 8 years; n = 17**				**9 to 14 years; n = 20**				**15 to 26 years; n = 18**			
	Sampling timepoint	Baseline	Post-dose 1	Pre-dose 2	Post-dose 2	Baseline	Post-dose 1	Pre-dose 2	Post-dose 2	Baseline	Post-dose 1	Pre-dose 3	Post-dose 3
No. of IgM secreting cells per 10^6 PBMC	25% Percentile	0	0	0	0	0	1	0	0	0	1	0	0
	Median	0	1	0	0	0	3	0	0	1	2	0	0
	75% Percentile	1	2	1	1	1	4	1	3	2	4	1	1

Table S3. Frequencies of HPV 16/18-specific IgG Bmem after Gardasil 9 vaccination

A: HPV 16 pooled

	**All ages**	**4 to 26 years; n = 53**			
	Sampling timepoint	Baseline	Post-dose 1	Pre-dose 2/3	Post-dose 2/3
% in total IgG memory B cells	25% Percentile	0.02	0.02	0.06	0.30
	Median	0.03	0.03	0.09	0.58
	75% Percentile	0.06	0.06	0.15	1.22

B: HPV 18 pooled

	**All ages**	**4 to 26 years; n = 53**			
	Sampling timepoint	Baseline	Post-dose 1	Pre-dose 2/3	Post-dose 2/3
% in total IgG memory B cells	25% Percentile	0.01	0.02	0.04	0.20
	Median	0.03	0.03	0.07	0.34
	75% Percentile	0.05	0.05	0.13	0.75

C: HPV 16 age-stratified

	**Age stratified**	**4 to 8 years; n = 18**				**9 to 14 years; n = 19**				**15 to 26 years; n = 16**			
	Sampling timepoint	Baseline	Post-dose 1	Pre-dose 2	Post-dose 2	Baseline	Post-dose 1	Pre-dose 2	Post-dose 2	Baseline	Post-dose 1	Pre-dose 3	Post-dose 3
% in total IgG memory B cells	25% Percentile	0.02	0.02	0.09	0.39	0.03	0.02	0.04	0.30	0.01	0.02	0.05	0.21
	Median	0.03	0.04	0.14	0.68	0.04	0.03	0.08	0.58	0.03	0.03	0.07	0.46
	75% Percentile	0.06	0.06	0.16	0.99	0.06	0.05	0.15	1.62	0.05	0.08	0.10	1.21

D: HPV 18 age-stratified

	**Age stratified**	**4 to 8 years; n = 18**				**9 to 14 years; n = 19**				**15 to 26 years; n = 16**			
	Sampling timepoint	Baseline	Post-dose 1	Pre-dose 2	Post-dose 2	Baseline	Post-dose 1	Pre-dose 2	Post-dose 2	Baseline	Post-dose 1	Pre-dose 3	Post-dose 3
% in total IgG memory B cells	25% Percentile	0.02	0.02	0.06	0.27	0.03	0.02	0.04	0.21	0.01	0.01	0.03	0.14
	Median	0.03	0.04	0.10	0.34	0.04	0.02	0.07	0.57	0.02	0.03	0.05	0.20
	75% Percentile	0.05	0.07	0.16	0.53	0.06	0.04	0.10	0.86	0.04	0.08	0.07	0.57

Table S4. Frequencies of ex vivo activated Tfh cells following Gardasil 9 vaccination

A: Total Tfh pooled

	**All ages**	**4 to 26 years**			
		**n = 44**	**n = 44**	**n = 51**	**n = 51**
	Sampling timepoint	Baseline	Post-dose 1	Pre-dose 2/3	Post-dose 2/3
% ICOS+PD-1++ in total Tfh cells	25% Percentile	0.69	0.80	0.88	1.44
	Median	1.18	1.41	1.36	2.25
	75% Percentile	1.83	2.27	1.91	3.78

B: Tfh1 pooled

	**All ages**	**4 to 26 years**			
		**n = 44**	**n = 44**	**n = 51**	**n = 51**
	Sampling timepoint	Baseline	Post-dose 1	Pre-dose 2/3	Post-dose 2/3
% ICOS+PD-1++ in Tfh1 cells	25% Percentile	0.73	1.02	0.98	1.96
	Median	1.24	1.59	1.50	3.40
	75% Percentile	2.37	2.89	3.28	4.72

C: Tfh2 pooled

	**All ages**	**4 to 26 years**			
		**n = 44**	**n = 44**	**n = 51**	**n = 51**
	Sampling timepoint	Baseline	Post-dose 1	Pre-dose 2/3	Post-dose 2/3
% ICOS+PD-1++ in Tfh2 cells	25% Percentile	0.65	0.82	0.77	1.64
	Median	1.16	1.58	1.18	2.18
	75% Percentile	1.79	2.45	1.89	3.51

D: Tfh17 pooled

	**All ages**	**4 to 26 years**			
		**n = 44**	**n = 44**	**n = 51**	**n = 51**
	Sampling timepoint	Baseline	Post-dose 1	Pre-dose 2/3	Post-dose 2/3
% ICOS+PD-1++ in Tfh17 cells	25% Percentile	0.53	0.47	0.72	0.63
	Median	0.86	1.09	1.21	1.29
	75% Percentile	1.79	1.71	1.52	2.27

E: Total Tfh age-stratified

	**Age stratified**	**4 to 8 years; n = 18**				**9 to 14 years; n = 19**				**15 to 26 years; n = 16**			
		**n = 20**	**n = 20**	**n = 19**	**n = 19**	**n = 16**	**n = 16**	**n = 16**	**n = 16**	**n = 8**	**n = 8**	**n = 16**	**n = 16**
	Sampling timepoint	Baseline	Post-dose 1	Pre-dose 2	Post-dose 2	Baseline	Post-dose 1	Pre-dose 2	Post-dose 2	Baseline	Post-dose 1	Pre-dose 3	Post-dose 3
% ICOS+PD-1++ in total Tfh cells	25% Percentile	1.18	1.62	1.39	2.15	0.71	0.65	0.92	2.03	0.16	0.19	0.66	0.95
	Median	1.66	2.24	1.91	3.23	1.17	0.99	1.56	3.27	0.68	0.65	0.90	1.41
	75% Percentile	2.13	2.81	2.61	3.87	1.36	1.54	1.80	4.17	0.95	1.04	1.07	1.68

Continued next page

F: Tfh1 age-stratified

	**Age stratified**	**4 to 8 years; n = 18**				**9 to 14 years; n = 19**				**15 to 26 years; n = 16**			
		**n = 20**	**n = 20**	**n = 19**	**n = 19**	**n = 16**	**n = 16**	**n = 16**	**n = 16**	**n = 8**	**n = 8**	**n = 16**	**n = 16**
	Sampling timepoint	Baseline	Post-dose 1	Pre-dose 2	Post-dose 2	Baseline	Post-dose 1	Pre-dose 2	Post-dose 2	Baseline	Post-dose 1	Pre-dose 3	Post-dose 3
% ICOS+PD-1++ in Tfh1 cells	25% Percentile	1.23	1.59	1.50	2.48	0.66	0.56	1.09	3.58	0	0.06	0.66	1.33
	Median	2.12	2.72	2.11	3.40	1.14	1.17	1.85	4.61	0.67	0.81	0.96	2.07
	75% Percentile	3.27	4.14	3.43	4.29	2.21	1.69	3.58	6.15	0.80	1.39	1.34	3.24

G: Tfh2 age-stratified

	**Age stratified**	**4 to 8 years; n = 18**				**9 to 14 years; n = 19**				**15 to 26 years; n = 16**			
		**n = 20**	**n = 20**	**n = 19**	**n = 19**	**n = 16**	**n = 16**	**n = 16**	**n = 16**	**n = 8**	**n = 8**	**n = 16**	**n = 16**
	Sampling timepoint	Baseline	Post-dose 1	Pre-dose 2	Post-dose 2	Baseline	Post-dose 1	Pre-dose 2	Post-dose 2	Baseline	Post-dose 1	Pre-dose 3	Post-dose 3
% ICOS+PD-1++ in Tfh2 cells	25% Percentile	1.06	1.67	0.84	2.13	0.77	0.81	0.77	1.80	0.31	0.33	0.44	0.90
	Median	1.54	2.50	1.51	2.91	0.98	1.12	1.15	2.83	0.60	0.77	0.82	1.44
	75% Percentile	2.69	3.12	2.38	3.93	1.48	1.60	1.91	3.99	1.023	1.64	1.18	2.03

H: Tfh17 age-stratified

	**Age stratified**	**4 to 8 years; n = 18**				**9 to 14 years; n = 19**				**15 to 26 years; n = 16**			
		**n = 20**	**n = 20**	**n = 19**	**n = 19**	**n = 16**	**n = 16**	**n = 16**	**n = 16**	**n = 8**	**n = 8**	**n = 16**	**n = 16**
	Sampling timepoint	Baseline	Post-dose 1	Pre-dose 2	Post-dose 2	Baseline	Post-dose 1	Pre-dose 2	Post-dose 2	Baseline	Post-dose 1	Pre-dose 3	Post-dose 3
% ICOS+PD-1++ in Tfh17 cells	25% Percentile	0.79	0.82	1.09	1.17	0.00	0.40	0.72	0.59	0.08	0.10	0.51	0.39
	Median	1.19	1.49	1.35	2.15	0.55	0.75	1.25	1.87	0.62	0.50	0.83	0.67
	75% Percentile	2.44	1.76	2.19	2.93	1.57	1.95	1.5	2.82	0.98	1.00	1.18	1.43

Table S5. Frequencies of activated HPV 16 specific Tfh cells after Gardasil 9 vaccination

A: OX40+CD25+ pooled

	**All ages**	**4 to 26 years**			
		**n = 40**	**n = 40**	**n = 41**	**n = 41**
	Sampling timepoint	Baseline	Post-dose 1	Pre-dose 2/3	Post-dose 2/3
% OX40+CD25+ in total Tfh cells	25% Percentile	0.00	0.00	0.10	0.14
	Median	0.03	0.10	0.48	0.38
	75% Percentile	0.14	0.40	1.27	1.45

B: OX40+PD-L1+ pooled

	**All ages**	**4 to 26 years**			
		**n = 40**	**n = 40**	**n = 41**	**n = 41**
	Sampling timepoint	Baseline	Post-dose 1	Pre-dose 2/3	Post-dose 2/3
% OX40+PD-L1+ in total Tfh cells	25% Percentile	0.00	0.00	0.05	0.08
	Median	0.00	0.04	0.51	0.42
	75% Percentile	0.08	0.13	2.28	1.70

C: PD-L1+CD25+ pooled

	**All ages**	**4 to 26 years**			
		**n = 40**	**n = 40**	**n = 41**	**n = 41**
	Sampling timepoint	Baseline	Post-dose 1	Pre-dose 2/3	Post-dose 2/3
% PD-L1+CD25+ in total Tfh cells	25% Percentile	0.00	0.02	0.12	0.24
	Median	0.12	0.29	0.45	0.81
	75% Percentile	0.47	1.03	1.97	2.34

D: OX40+CD25+ age-stratified

	**Age stratified**	**4 to 8 years; n = 18**				**9 to 14 years; n = 19**				**15 to 26 years; n = 16**			
		**n = 15**	**n = 15**	**n = 15**	**n = 15**	**n = 12**	**n = 12**	**n = 12**	**n = 12**	**n = 13**	**n = 13**	**n = 14**	**n = 14**
	Sampling timepoint	Baseline	Post-dose 1	Pre-dose 2	Post-dose 2	Baseline	Post-dose 1	Pre-dose 2	Post-dose 2	Baseline	Post-dose 1	Pre-dose 3	Post-dose 3
% OX40+CD25+ in total Tfh cells	25% Percentile	0.00	0.02	0.05	0.10	0.00	0.00	0.18	0.21	0.03	0.00	0.21	0.21
	Median	0.00	0.07	0.16	0.17	0.01	0.13	0.76	0.52	0.12	0.15	0.77	0.78
	75% Percentile	0.06	0.32	0.67	0.52	0.13	0.63	3.54	1.57	0.66	0.52	2.21	1.85

Continued next page

E: OX40+PD-L1+ age-stratified

	Age stratified	4 to 8 years; n = 18				9 to 14 years; n = 19				15 to 26 years; n = 16			
		**n = 15**	**n = 15**	**n = 15**	**n = 15**	**n = 12**	**n = 12**	**n = 12**	**n = 12**	**n = 13**	**n = 13**	**n = 14**	**n = 14**
	Sampling timepoint	Baseline	Post-dose 1	Pre-dose 2	Post-dose 2	Baseline	Post-dose 1	Pre-dose 2	Post-dose 2	Baseline	Post-dose 1	Pre-dose 3	Post-dose 3
% OX40+PD-L1+ in total Tfh cells	25% Percentile	0.00	0.00	0.00	0.00	0.00	0.01	0.06	0.04	0.00	0.01	0.12	0.30
	Median	0.00	0.00	0.07	0.23	0.00	0.07	1.10	1.27	0.05	0.11	1.39	1.12
	75% Percentile	0.05	0.05	0.51	0.53	0.04	0.18	4.31	1.98	0.51	0.37	3.95	1.97

F: PD-L1+CD25+ age-stratified

	Age stratified	4 to 8 years; n = 18				9 to 14 years; n = 19				15 to 26 years; n = 16			
		**n = 15**	**n = 15**	**n = 15**	**n = 15**	**n = 12**	**n = 12**	**n = 12**	**n = 12**	**n = 13**	**n = 13**	**n = 14**	**n = 14**
	Sampling timepoint	Baseline	Post-dose 1	Pre-dose 2	Post-dose 2	Baseline	Post-dose 1	Pre-dose 2	Post-dose 2	Baseline	Post-dose 1	Pre-dose 3	Post-dose 3
% PD-L1+CD25+ in total Tfh cells	25% Percentile	0.00	0.00	0.00	0.09	0.05	0.13	0.04	0.78	0.00	0.17	0.40	0.41
	Median	0.00	0.21	0.15	0.27	0.22	0.34	0.90	1.65	0.15	0.30	1.51	1.59
	75% Percentile	0.23	0.41	0.35	0.81	0.64	1.23	5.26	4.21	1.11	1.12	3.53	4.64

Table S6. Frequencies of activated HPV 18 specific Tfh cells after Gardasil 9 vaccination

A: OX40+CD25+ pooled

	**All ages**	**4 to 26 years**			
		**n = 39**	**n = 39**	**n = 40**	**n = 40**
	Sampling timepoint	Baseline	Post-dose 1	Pre-dose 2/3	Post-dose 2/3
% OX40+CD25+ in total Tfh cells	25% Percentile	0.00	0.07	0.00	0.02
	Median	0.08	0.15	0.42	0.34
	75% Percentile	0.22	0.89	0.89	1.30

B: OX40+PD-L1+ pooled

	**All ages**	**4 to 26 years**			
		**n = 40**	**n = 40**	**n = 41**	**n = 41**
	Sampling timepoint	Baseline	Post-dose 1	Pre-dose 2/3	Post-dose 2/3
% OX40+PD-L1+ in total Tfh cells	25% Percentile	0.00	0.00	0.05	0.12
	Median	0.04	0.14	0.42	0.53
	75% Percentile	0.09	0.33	1.84	1.71

C: PD-L1+CD25+ pooled

	**All ages**	**4 to 26 years**			
		**n = 39**	**n = 39**	**n = 40**	**n = 40**
	Sampling timepoint	Baseline	Post-dose 1	Pre-dose 2/3	Post-dose 2/3
% PD-L1+CD25+ in total Tfh cells	25% Percentile	0.00	0.19	0.02	0.34
	Median	0.05	0.47	0.35	1.09
	75% Percentile	0.47	1.67	1.04	3.93

D: OX40+CD25+ age-stratified

	**Age stratified**	**4 to 8 years; n = 18**				**9 to 14 years; n = 19**				**15 to 26 years; n = 16**			
		**n = 15**	**n = 15**	**n = 15**	**n = 15**	**n = 12**	**n = 12**	**n = 12**	**n = 12**	**n = 12**	**n = 12**	**n = 13**	**n = 13**
	Sampling timepoint	Baseline	Post-dose 1	Pre-dose 2	Post-dose 2	Baseline	Post-dose 1	Pre-dose 2	Post-dose 2	Baseline	Post-dose 1	Pre-dose 3	Post-dose 3
% OX40+CD25+ in total Tfh cells	25% Percentile	0.00	0.00	0.00	0.00	0.00	0.08	0.00	0.05	0.00	0.08	0.00	0.11
	Median	0.08	0.08	0.22	0.19	0.06	0.15	0.70	0.69	0.06	0.15	0.74	0.43
	75% Percentile	0.27	0.98	0.47	1.16	0.22	0.81	1.19	1.75	0.22	0.81	1.63	1.61

Continued next page

E: OX40+PD-L1+ age-stratified

	**Age stratified**	**4 to 8 years; n = 18**				**9 to 14 years; n = 19**				**15 to 26 years; n = 16**			
		**n = 15**	**n = 15**	**n = 15**	**n = 15**	**n = 12**	**n = 12**	**n = 12**	**n = 12**	**n = 12**	**n = 12**	**n = 14**	**n = 14**
	Sampling timepoint	Baseline	Post-dose 1	Pre-dose 2	Post-dose 2	Baseline	Post-dose 1	Pre-dose 2	Post-dose 2	Baseline	Post-dose 1	Pre-dose 3	Post-dose 3
% OX40+PD-L1+ in total Tfh cells	25% Percentile	0.00	0.00	0.01	0.07	0.00	0.05	0.00	0.12	0.00	0.00	0.24	0.19
	Median	0.00	0.00	0.20	0.23	0.05	0.16	0.70	1.17	0.04	0.29	1.48	0.60
	75% Percentile	0.09	0.18	0.42	1.12	0.10	0.62	1.80	1.99	0.18	0.40	4.36	1.86

F: PD-L1+CD25+ age-stratified

	**Age stratified**	**4 to 8 years; n = 18**				**9 to 14 years; n = 19**				**15 to 26 years; n = 16**			
		**n = 15**	**n = 15**	**n = 15**	**n = 15**	**n = 12**	**n = 12**	**n = 12**	**n = 12**	**n = 12**	**n = 12**	**n = 13**	**n = 13**
	Sampling timepoint	Baseline	Post-dose 1	Pre-dose 2	Post-dose 2	Baseline	Post-dose 1	Pre-dose 2	Post-dose 2	Baseline	Post-dose 1	Pre-dose 3	Post-dose 3
% PD-L1+CD25+ in total Tfh cells	25% Percentile	0.00	0.05	0.00	0.07	0.01	0.32	0.11	0.38	0.01	0.32	0.15	0.40
	Median	0.00	0.34	0.11	0.39	0.09	0.60	0.86	1.63	0.09	0.60	0.86	1.26
	75% Percentile	0.50	1.73	0.34	1.52	0.45	1.49	1.67	4.47	0.45	1.49	1.74	4.33

Table S7. Tfh ex vivo antibody panel

Antibody	Clone	Isotype	Company	Catalogue number
AlexaFluor (AF) 700 mouse anti-human CD4	RPA-T4	mIgG1	BioLegend	300526
PECy7 anti-human/mouse/rat CD278 (ICOS)	C398.4A	Armenian hamster IgG	BioLegend	313520
PE mouse anti-human CD279 (PD-1), eBioscience	eBioJ105	mIgG1	Thermo Fisher Scientific	12-2799-42
PerCPCy5.5 mouse anti-human CXCR5	J252D4	mIgG1	BioLegend	356910
APC/Cyanine7 anti-human CD183 (CXCR3)	G025H7	mIgG1	BioLegend	353722
Brilliant Violet (BV) 605 mouse anti-human CD45RO	UCHL1	mIgG1	BioLegend	304238
BV421 mouse anti-human CCR6 (CD196)	11A9	mIgG1	BD Biosciences	562515
V500 Zombie Aqua™ Fixable Viability dye	-	-	BioLegend	423102

Table S8. Tfh AIM antibody panel

Antibody	Clone	Isotype	Company	Catalogue number
Brilliant Violet (BV) 650 Mouse Anti-Human CD134 (OX40)	Ber-ACT35 (ACT35)	mIgG1	BD Biosciences	563658
BV786 mouse anti-human CD25	M-A251	mIgG1	BD Biosciences	563701
AlexaFluor (AF) 700 mouse anti-human CD4	RPA-T4	mIgG1	BioLegend	300526
PerCPCy5.5 mouse anti-human CXCR5	J252D4	mIgG1	BioLegend	356910
PE mouse anti-human CD274 (B7-H1, PD-L1)	29E.2A3	mIgG2b	BioLegend	329706
PECy7 mouse anti-human CD278 (ICOS)	C398.4A	Armenian hamster IgG	BioLegend	313520
BV605 mouse anti-human CD45RO	UCHL1	mIgG1	BioLegend	304238
PE-CF594 mouse anti-human FoxP3	236A/E7	mIgG1	BD Biosciences	563955
V500 mouse anti-human CD14	M5E2	mIgG2a	BD Biosciences	561391
V500 Zombie Aqua™ Fixable Viability dye	-	-	BioLegend	423102
